# Amplicon targeted resequencing for *SLC2A9* and *SLC22A12* identified novel mutations in hypouricemia subjects

**DOI:** 10.1002/mgg3.722

**Published:** 2019-05-26

**Authors:** Zhaowei Zhou, Ke Wang, Juan Zhou, Can Wang, Xinde Li, Lingling Cui, Lin Han, Zhen Liu, Wei Ren, Xuefeng Wang, Keke Zhang, Zhiqiang Li, Dun Pan, Changgui Li, Yongyong Shi

**Affiliations:** ^1^ Bio‐X Institutes, Key Laboratory for the Genetics of Developmental and Neuropsychiaric Disorders (Ministry of Education) Shanghai Jiao Tong University Shanghai P.R. China; ^2^ Qingdao Key Laboratory of Gout The Affiliated Hospital of Qingdao University Qingdao P.R. China; ^3^ Shandong Provincial Key Laboratory of Metabolic Disease The Affiliated Hospital of Qingdao University Qingdao P.R. China; ^4^ Metabolic Disease Institute Qingdao University Qingdao P.R. China; ^5^ The Department of Endocrinology and Metabolism The Affiliated Hospital of Qingdao University Qingdao P.R. China; ^6^ Biomedical Sciences Institute, the Qingdao Branch of SJTU Bio‐X Institutes, Qingdao University Qingdao P.R. China

**Keywords:** hypouricemia, next‐generation sequencing (NGS), rare variants, *SLC22A12*, *SLC2A9*

## Abstract

**Background:**

To identify potential causative mutations in *SLC2A9* and *SLC22A12* that lead to hypouricemia or hyperuricemia (HUA).

**Methods:**

Targeted resequencing of whole exon regions of *SLC2A9* and *SLC22A12* was performed in three cohorts of 31 hypouricemia, 288 HUA and 280 normal controls.

**Results:**

A total of 84 high‐quality variants were identified in these three cohorts. Eighteen variants were nonsynonymous or in splicing region, and then included in the following association analysis. For common variants, no significant effects on hypouricemia or HUA were identified. For rare variants, six single nucleotide variations (SNVs) p.T21I and p.G13D in *SLC2A9*, p.W50fs, p.Q382L, p.V547L and p.E458K in *SLC22A12,* occurred in totally six hypouricemia subjects and were absent in HUA and normal controls. Allelic and genotypic frequency distributions of the six SNVs differed significantly between the hypouricemia and normal controls even after multiple testing correction, and p.G13D in *SLC2A9* and p.V547L in *SLC22A12* were newly reported. All these mutations had no significant effects on HUA susceptibility, while the gene‐based analyses substantiated the significant results on hypouricemia.

**Conclusion:**

Our study first presents a comprehensive mutation spectrum of hypouricemia in a large Chinese cohort.

## 1 INTRODUCTION

Hypouricemia, conventionally defined as serum uric acid (SUA) concentration ≤2 mg/dl (Bordier et al., 2004), is not widely recognized in clinical practice. In fact, SUA performs the important physiological function of oxidative defense in human body, which is related to prolonged longevity and decreased age‐specific cancer incidence (Ames, Cathcart, Schwiers, & Hochstein, 1981). Although the accurate biological mechanisms remain unknown, growing researches emerge to provide evidence that lower SUA leads to varied pathophysiological conditions: hypouricemia has a higher incidence of acute kidney injuries (AKI) (Ichida et al., 2004), urolithiasis (Ichida et al., 2004), and composite cardiovascular events (Essex et al., 2017), and it is also associated with higher all‐cause mortality in hemodialysis patients (Park et al., 2017), as well as a risk factor of neurodegeneration progression and a potential indicator of malnutrition (Tana, Ticinesi, Prati, & Nouvenne, 2018).

Hypouricemia is mainly caused by genetic defects of impaired renal tubular reabsorption (Enomoto et al., 2002; Matsuo et al., 2008) and/or reduced UA production (Sebesta & Stiburkova, 2018). Among them, hereditary renal hypouricemia (hRHUC) is a major type due to mutations of urate transporter URAT1 (encoded by *SLC22A12* [OMIM *607096] and classified as hRHUC 1 [OMIM #220150]) and URATv1 (encoded by *SLC2A9* [OMIM *606142] and classified as hRHUC 2 [OMIM #612076]) and is prone to exercise‐induced acute renal failure (EIARF) and urolithiasis especially in men (Ichida et al., 2008; Kaito et al., 2013). Recently, hRHUC patients complicated with chronic renal failure have been reported (Aksoy, Koyun, Ichida, Comak, & Akman, 2018a, 2018b; Claverie‐Martin et al., 2018) and the correlation of hypouricemia with reduced kidney function was also established in a large‐scale cross‐sectional population‐based study (Wakasugi et al., 2015). Therefore, recognizing people with very low SUA and providing prompt medical guidance is of critical importance to avoid renal adverse events (Bhasin et al., 2014). With the popularity of genetic testing methods, more hRHUC patients have been reported. Notably, most published literatures on hypouricemia focused on case report or case serials (Zhou et al., 2018), which lack essential statistics through comparison with normal controls and may result in false reports of causative mutations to hypouricemia, especially in the Han Chinese population. Moreover, most reported hRHUC patients were mainly diagnosed using traditional Sanger sequencing (Kim et al., 2015; Windpessl, Ritelli, Wallner, & Colombi, 2016) or locus‐specific polymerase chain reaction (PCR) reaction (Takagi, Omae, Makanga, Kawahara, & Inazu, 2013) for one gene *SLC22A12* or *SLC2A9*. Although sanger sequencing is the golden standard for DNA detection but is time‐consuming and laborious (Sommen & Van Camp, 2013), next‐generation sequencing (NGS) based targeted resequencing has evolved to correctly diagnose genetic diseases in a more cost‐effective and time‐saving mode (Adams & Eng, 2018; Sommen & Van Camp, 2013). Using the NGS method and incorporating the parent‐offspring trios, we successfully confirmed a hRHUC patient cosegregated with novel compound heterozygous mutations in *SLC22A12* (Zhou et al., 2018).

In this study, we aim to detect the mutations in three cohorts of hypouricemia, hyeruricemia (HUA) and normal controls using an amplicon‐targeted NGS method for both *SLC22A12* and *SLC2A9*, and conduct statistical analyses to further corroborate the mutations with real hypouricemia or HUA susceptibility in the Han Chinese population.

## 2 MATERIALS AND METHODS

### 2.1 Ethical compliance

This study was approved by the local ethics committee and conformed to the principles of the Declaration of Helsinki (World Medical, 2013). All recruited participants signed informed consent for biomedical and genetic analysis.

### 2.2 Study participants for sequencing

To detect true *SLC22A12/SLC2A9* mutations that lead to hypouricemia, we sequenced three cohorts of hypouricemia, HUA and normal controls in order to perform comparative analyses. Thirty‐one hypouricemia, 280 normal controls and 288 long‐term HUA individuals who had not yet developed gout were recruited from Qingdao Key Laboratory of Gout. Demographic and clinical indices were retrospectively reviewed. Reference ranges of SUA: children under 15 years of age and adult females, 2.0–5.7 mg/dl (120–342 µmol/L); adult males, 2.0–7.0 mg/dl (120–420 µmol/L) (Stiburkova et al., 2013). Hypouricemia was defined as SUA ≤2 mg/dl (120 µmol/L) irrespective of sex (Claverie‐Martin et al., 2018; Gibson, Sims, & Jimenez, 1976; Ichida et al., 2004). HUA was defined as SUA >7 mg/dl (420 µmol/L). In normal controls, SUA was confined to 3–6 mg/dl (180–360 µmol/L). Unfortunately, we could not get past histories such as onset of AKI and urolithiasis and types of medications that may influence SUA concentrations.

### 2.3 Amplicon targeted resequencing

Genomic DNA was prepared using LifeFeng Genomic DNA Purification Kit (Lifefeng Biotech Co., Ltd., Shanghai, China) and quality controlled using a NanoDrop 1000 Spectrophotometer (Thermo Scientific, United States), as described (Li et al., 2015). Primers along with standard Illumina PE adapters were designed to generate 46 amplicons harboring all exons and partial un‐translated regions (UTRs) of the *SLC22A12* and *SLC2A9*. The primer sequences and targeted regions can be seen in Data S2. Sequence libraries were prepared in a two‐staged PCR process. The PCR reagents and procedures were designed and supported by the Shanghai DYnastyGene Company, according to the manual. Each sample was then ligated with unique 8 bp index for sample‐specific barcoding, which allowed all samples to be mixed for library purification and sequencing in a single run. The size distribution of fragments was determined using 2100 Bioanalyzer and the High Sensitivity DNA kit (Agilent Technologies, United States). The well‐constructed library was then sequenced as 150 bp paired‐end reads on an Illumina Xten platform (Illumina, United States).

### 2.4 Variant calling and quality control

Sequence reads were demultiplexed according to each known amplicon start and end sequence that allowed no base mismatch. Sequence reads were clipped to remove adapters by Trimmomatic v 0.30. Clipped sequence reads were aligned to the human genome 19 (hg19) for *SLC2A9* (NC_000004.11, region: 9827848…10041872) and *SLC22A12* (NC_000011.9, region: 64358113…64369825) using maximal exact matches command of BWA and BAM file was generated using SAMtools. Indel realignment and base quality recalibration were implemented using IndelRealigner and BQSR included in the Genome Analysis Toolkit (GATK). Variants including single nucleotide polymorphisms and short insertions and deletions (Indels) were called by GATK HaplotypeCaller and were annotated with Annovar. To avoid false positives in the best way, we applied hard filtration with reads depth >100, mapping quality >30, base quality >30.

Variants were expressed in two forms of nucleotide changeand amino acid change according to reference sequences of *SLC2A9* (NM_020041.3; NP_064425.2) and *SLC22A12* (NM_144585.4; NP_653186.2).

### 2.5 Statistical analysis

Differences in demographic and clinical indices between pairwise groups were estimated using independent *t* test (quantitative traits) or chi‐square (χ^2^) test (qualitative traits) with SPSS 19.0. To assess the group representation, Hardy‐Weinberg equilibrium (HWE) was conducted in the normal controls. The χ^2^ test was used for association analyses with effect actions indicated by odds ratios (ORs) with 95% confidence intervals. Statistical analyses were performed using free‐charge SHEsis online platform (http://shesisplus.bio-x.cn/) (Shi & He, 2005). Haploview 4.2 was used to analyze pairwise linkage disequilibrium (LD) and haplotype distributions for the common variants. Two‐tailed p value of < 0.05 was considered statistically significant. The Bonferroni correction method was used for multiple testing, which would decrease the significance threshold (=0.05 divided by numbers of variants to be analyzed).

## 3 RESULTS

### 3.1 Clinical characteristics of the study participants

**Figure 1:**
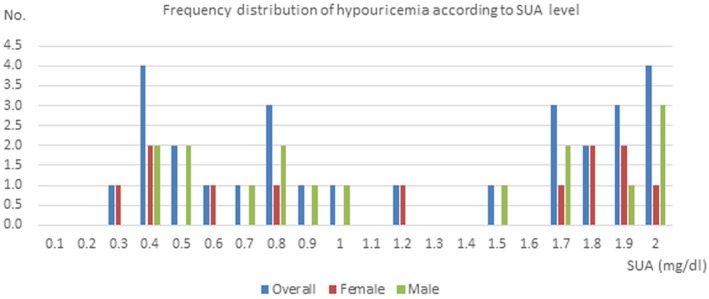
Frequency distribution of hypouricemia according to SUA level. Two SUA sections were concentrated in hypouricemia frequency with the first at 0.3–1.0 mg/dl and the second at 1.7–2.0 mg/dl. SUA, serum uric acid

The mean SUA values among hypouricemia, normal controls and HUA were 1.18 mg/dl, 4.47 mg/dl and 7.93 mg/dl, respectively. No significant difference was observed with respect to gender ratio, age, waist circumference and fasting glucose between pairwise groups (Table 1). Other demographic and clinical indices with significant difference are also summarized in Table 1. Next, we arranged the hypouricemia samples according to SUA values. Two SUA sections were concentrated in hypouricemia frequency with the first at 0.3–1.0 mg/dl and the second at 1.7–2.0 mg/dl (Figure 1).

**Table 1:** The demographic and clinical indices among hypouricemia (1), normal control (2) and HUA (3) groups

Indices	(1)	(2)	(3)	(1) vs. (2) p	(3) vs. (2) p
Male (%)	54.8	50.7	52.8	0.663	0.623
Age (years)	53.00 ± 21.12	59.27 ± 14.25	58.82 ± 17.20	0.116	0.742
WC (cm)	86.64 ± 13.93	92.09 ± 8.45	93.39 ± 10.34	0.086	0.161
HC (cm)	96.93 ± 8.80	102.32 ± 6.56	102.82 ± 8.59	**0.001**	0.507
BMI (kg/m^2^)	23.74 ± 3.94	25.31 ± 3.16	26.27 ± 3.85	0.05	**0.003**
SP (mmHg)	130.12 ± 20.64	138.57 ± 19.02	139.60 ± 20.95	**0.033**	0.564
DP (mmHg)	80.65 ± 11.19	82.98 ± 11.57	86.50 ± 12.22	0.328	**0.001**
Glucose (mmol/L)	5.66 ± 1.89	6.24 ± 2.25	6.03 ± 1.72	0.175	0.241
Triglyceride (mmol/L)	1.84 ± 1.66	1.52 ± 0.95	2.28 ± 1.94	0.296	**<0.001**
Cholesterol (mmol/L)	5.20 ± 1.03	5.87 ± 1.12	5.38 ± 1.09	**0.002**	**<0.001**
BUN (mmol/L)	5.56 ± 1.69	5.57 ± 1.21	6.05 ± 2.03	0.966	**0.002**
Creatinine (μmol/L)	68.10 ± 21.01	67.54 ± 15.24	85.44 ± 23.91	0.888	**<0.001**
SUA (mg/dl)	1.18 ± 0.62	4.47 ± 0.84	7.93 ± 0.96	**<0.001**	**<0.001**

Note

To convert SUA in mg/dL to μmol/L, multiply by 60.

Abbreviations: BMI, Body mass index; BUN, Blood urea nitrogen; HC, DP, Diastolic pressure; Hip circumference; HUA, hypouricemia or hyperuricemia; SP, Systolic pressure; SUA, Serum uric acid; WC, Waist circumference.

*p* < 0.05 as statistical significance and significant *p*‐values in bold.

### 3.2 Variants identification

A total of 84 high‐quality variants were called in the three cohorts including 17 nonsynonymous (15 missense, one frameshift and one stopgain), one splicing region variant, 18 synonymous mutations, 31 intron variants, three upstream or downstream variants and 14 UTR variants (Data S2). In total, 41 common variants (minor allele frequency (MAF) >0.01) and 43 rare variants (MAF <0.01) were identified. On the whole, 17 were newly reported (Data S2).

### 3.3 Association analyses of the variants with hypouricemia and HUA

**Figure 2:**
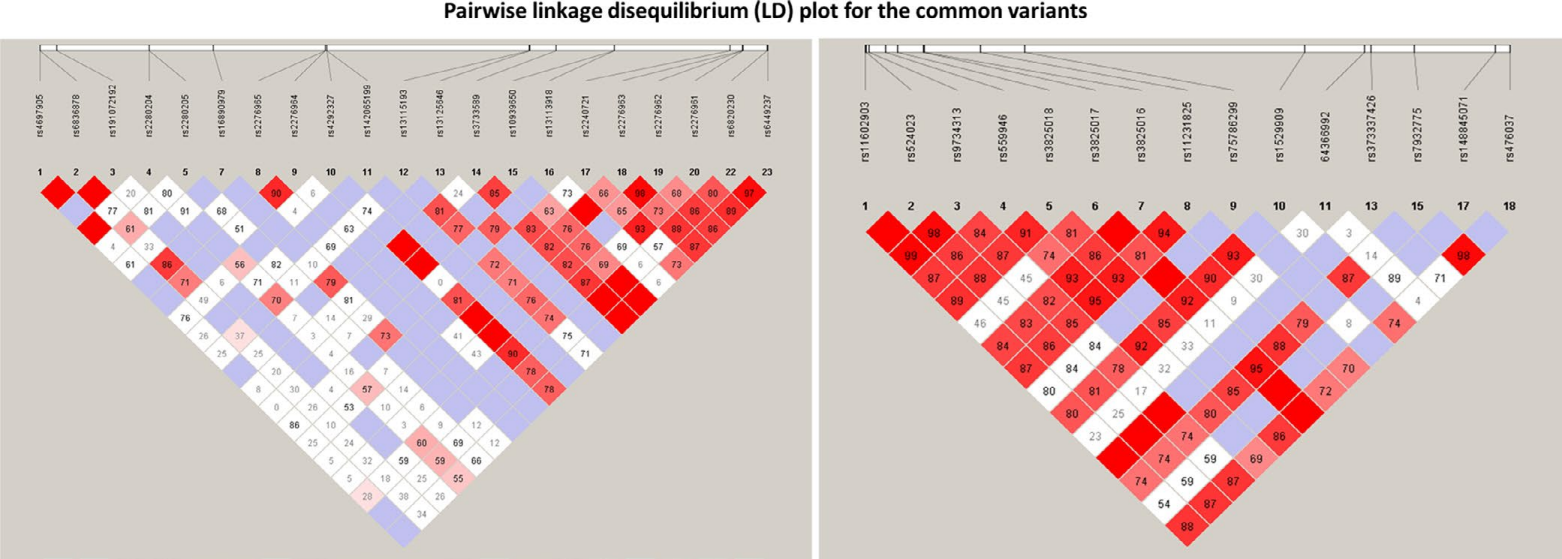
Pairwise linkage disequilibrium (LD) plot for the common variants. (a) demonstrates the strength of the pairwise LD based on D′ in *SLC2A9*, and numbers represent the value of D′ expressed as a percentage. The blanks represent D′ = 1. (b) demonstrates the strength of the pairwise LD based on D’ in *SLC22A12*, and numbers represent the value of D′ expressed as a percentage. The blanks represent D′ = 1

Seeking for potential hypouricemia causative mutations, we selected the 17 nonsynonymous mutations and the one splicing region variant and performed comparative analyses to confirm the disease susceptibility (Table 2). Thus, the significance threshold should be a *p* value of 0.003 (=0.05/18). Among them, common variant rs3733591 failed to reach HWE with the *p* value being 0.0002 in the healthy controls and then were excluded from analysis. For the reported common variant rs16890979, T allele conferred substantial risk for hypouricemia although the allelic association just showed a marginal effect (OR = 3.5085, *p* = 0.0532). However, its genotypic difference reached nominal significance (*p* = 0.0107). These associations did not reach statistical significance in terms of Bonferroni correction. Comparing the HUA cohort with the normal controls, rs16890979 T allele conferred no significant effect on HUA susceptibility (OR = 0.7263, *p* = 0.5546). For the other reported common variants rs2280205, rs6820230 and rs2276961, the effects on hypouricemia or HUA were not identified in our datasets. Pairwise LD analyses indicated that the common variants existed in one haplotype block in *SLC2A9* and *SLC22A12*, respectively (Figure 2) and no significant haplotype distribution was found (data not shown).

**Table 2:** Allele and genotype distributions among hypouricemia (1), normal control (2) and hyperuricemia (3) groups

CHR	BP	Variants	Allele and genotype	(1)	(2)	(3)	(1) vs. (2) OR	(1) vs. (2) *p*	(3) vs. (2) OR	(3) vs. (2) *p*	HWE in (2)	Novel or not
chr4	9909923	*SLC2A9*: c.G1049A/p.P350L	G	45(0.726)	402(0.718)	409(0.710)	0.96	0.8949	1.039	0.7715	0.3318	rs2280205
			A	17(0.274)	158(0.282)	167(0.290)	0.53 ~ 1.73		0.80 ~ 1.34			
			G/G	15(0.484)	141(0.504)	134(0.465)		0.6811		0.2368		
			G/A	15(0.484)	120(0.429)	141(0.490)						
			A/A	1(0.032)	19(0.068)	13(0.045)						
chr4	9922167	*SLC2A9*: c.C844T/ p.V282I	C	59(0.952)	552(0.986)	570(0.990)	3.51	0.0533	0.73	0.5546	0.8084	rs16890979
			T	3(0.048)	8(0.014)	6(0.010)	0.91 ~ 13.59		0.25 ~ 2.11			
			C/C	29(0.935)	272(0.971)	282(0.979)		0.0107		0.5521		
			C/T	1(0.032)	8(0.029)	6(0.021)						
			T/T	1(0.032)	0(0.000)	0(0.000)						
chr4	10020615	*SLC2A9*: c.A233G/p.V78A	A	62(1.000)	559(0.998)	575(0.998)	—	0.7391	0.97	0.9841	0.9761	rs907584363
			G	0(0.000)	1(0.002)	1(0.002)			0.06 ~ 15.58			
			A/A	31(1.000)	279(0.996)	287(0.997)		0.7389		0.9841		
			A/G	0(0.000)	1(0.004)	1(0.003)						
chr4	10020618	*SLC2A9*: c.A230C/p.V77G	A	62(1.000)	560(1.000)	575(0.998)	—	—	—	0.324	1	rs183263293
			C	0(0.000)	0(0.000)	1(0.002)						
			A/A	31(1.000)	280(1.000)	287(0.997)		—		0.3237		
			A/C	0(0.000)	0(0.000)	1(0.003)						
chr4	10022981	*SLC2A9*: c.C73T/p.G25R	C	35(0.565)	277(0.495)	288(0.500)	0.76	0.2964	0.30	0.8567	0.9064	rs2276961
			T	27(0.435)	283(0.505)	288(0.500)	0.45 ~ 1.28		0.78 ~ 1.24			
			C/C	11(0.355)	69(0.246)	63(0.219)		0.4227		0.2839		
			C/T	13(0.419)	139(0.496)	162(0.562)						
			T/T	7(0.226)	72(0.257)	63(0.219)						
chr4	10022992	*SLC2A9*: c.G62A/p.T21I	G	61(0.984)	560(1.000)	576(1.000)	—	**0.0026**	—	—	1	rs748372830
			A	1(0.016)	0(0.000)	0(0.000)						
			A/G	1(0.032)	0(0.000)	0(0.000)		**0.0026**		—		
			G/G	30(0.968)	280(1.000)	288(1.000)						
chr4	10023016	*SLC2A9*: c.C38T/p.G13D	C	61(0.984)	560(1.000)	576(1.000)	—	**0.0026**	—	—	1	Novel
			T	1(0.016)	0(0.000)	0(0.000)						
			C/C	30(0.968)	280(1.000)	288(1.000)		**0.0026**		—		
			C/T	1(0.032)	0(0.000)	0(0.000)						
chr4	10027542	*SLC2A9*: c.C49T/p.A17T *	C	58(0.935)	519(0.927)	533(0.925)	0.87	0.8019	1.02	0.9262	0.6591	rs6820230
			T	4(0.065)	41(0.073)	43(0.075)	0.30 ~ 2.52		0.65 ~ 1.59			
			C/C	27(0.871)	240(0.857)	246(0.854)		0.9333		0.9943		
			C/T	4(0.129)	39(0.139)	41(0.142)						
			T/T	0(0.000)	1(0.004)	1(0.003)						
chr11	64359297	*SLC22A12*: c.G269A/p.R90H	G	62(1.000)	558(0.996)	576(1.000)	—	0.6374	—	0.1512	0.9522	rs121907896
			A	0(0.000)	2(0.004)	0(0.000)						
			G/G	31(1.000)	278(0.993)	288(1.000)		0.6369		0.1508		
			G/A	0(0.000)	2(0.007)	0(0.000)						
chr11	64360303	*SLC22A12*: c.A455G/p.Y152C	A	62(1.000)	559(0.998)	576(1.000)	—	0.7391	—	0.3103	0.9761	Novel
			G	0(0.000)	1(0.002)	0(0.000)						
			A/A	31(1.000)	279(0.996)	288(1.000)		0.7389		0.31		
			A/G	0(0.000)	1(0.004)	0(0.000)						
chr11	64360355	*SLC22A12*: c.506 + 1G>A	G	62(1.000)	560(1.000)	575(0.998)	—	—	—	0.324	1	rs58174038
			A	0(0.000)	0(0.000)	1(0.002)						
			G/G	31(1.000)	280(1.000)	287(0.997)		—		0.324		
			G/A	0(0.000)	0(0.000)	1(0.003)						
chr11	64361134	*SLC22A12*: c.G689A/p.R230Q	G	62(1.000)	559(0.998)	576(1.000)	—	0.7391	—	0.3103	0.9761	rs759297223
			A	0(0.000)	1(0.002)	0(0.000)						
			G/G	31(1.000)	279(0.996)	288(1.000)		0.7389		0.3101		
			G/A	0(0.000)	1(0.004)	0(0.000)						
chr11	64361219	*SLC22A12*: c.G774A/p.W258X	G	62(1.000)	559(0.998)	576(1.000)	—	0.7391	—	0.3103	0.9761	rs121907892
			A	0(0.000)	1(0.002)	0(0.000)						
			G/G	31(1.000)	279(0.996)	288(1.000)		0.7389		0.3101		
			G/A	0(0.000)	1(0.004)	0(0.000)						
chr11	64367925	*SLC22A12*: c.G1372A/p.E458K	G	60(0.968)	560(1.000)	576(1.000)	—	**2.11E^−05^**	—	—	1	rs747742344
			A	2(0.032)	0(0.000)	0(0.000)						
			G/G	30(0.968)	280(1.000)	288(1.000)		**0.0026**		—		
			G/A	1(0.032)	0(0.000)	0(0.000)						
chr11	64359177	*SLC22A12*: c.149delG/p.W50fs	G	60(0.968)	560(1.000)	576(1.000)	—	**2.11E^−05^**	—	—	1	rs752156476
			—	2(0.032)	0(0.000)	0(0.000)						
			G/G	29(0.935)	280(1.000)	288(1.000)		**2.05E^−05^**		—		
			G/—	2(0.065)	0(0.000)	0(0.000)						
chr11	64367222	*SLC22A12*: c.A1145T/p.Q382L	A	61(0.984)	560(1.000)	576(1.000)	—	**0.0026**	—	—	1	rs765990518
			T	1(0.016)	0(0.000)	0(0.000)		** **				
			A/A	30(0.968)	280(1.000)	288(1.000)		**0.0026**		—		
			A/T	1(0.032)	0(0.000)	0(0.000)		** **				
chr11	64369000	*SLC22A12*: c.G1639C/p.V547L	G	61(0.984)	560(1.000)	576(1.000)	—	**0.0026**	—	—	1	Novel
			C	1(0.016)	0(0.000)	0(0.000)		** **				
			C/G	1(0.032)	0(0.000)	0(0.000)		**0.0026**		—		
			G/G	30(0.968)	280(1.000)	288(1.000)						

Note

BP, base position; CHR, chromosome; HWE, Hardy‐Weinberg equilibrium; OR, odds ratio; “—” means uncalculated; *p* < 0.003 as statistical significance and significant *p*‐values in bold. BP was determined using reference sequences of S*LC2A9* (NC_000004.11, region: 9827848…10041872) and *SLC22A12* (NC_000011.9, region: 64358113…64369825) and variants were expressed in two forms of nucleotide change and amino acid change according to reference sequences of *SLC2A9* (NM_020041.3; NP_064425.2) and *SLC22A12* (NM_144585.4; NP_653186.2).

* Denotes the variant annotated by *SLC2A9* (NM_001001290.1; NP_001001290.1). For each variant, the allele in the first line was the reference allele and the second line was the altered allele that the reported OR correlates with.

We then focused on the rare mutations. Six single nucleotide variations (SNVs) *SLC2A9*: p.T21I, *SLC2A9*: p.G13D, *SLC22A12*: p.W50fs, *SLC22A12*: p.Q382L, *SLC22A12*: p.V547L and *SLC22A12*: p.E458K occurred in six hypouricemia subjects (Table 3). Patient 1 encoded with QQY23 had SUA of 0.63 mg/dl and carried a heterozygous *SLC2A9*: p.T21I. Patient 2 encoded with 17QD5230 had SUA of 1.57 mg/dl and carried a heterozygous *SLC2A9*: p.G13D which was first reported in this study. Patient 3 and 4 encoded with QLY630 and N2401 had comparable SUA level of ≈1.9 mg/dl and carried the same heterozygous mutant *SLC22A12*: p.W50fs. Patient 5 encoded with 17QD3912 had SUA of 1.77 mg/dl and carried compound heterozygous *SLC22A12*: p.Q382L and *SLC22A12*: p.V547L. Patient 6 encoded with 17QD2146 had SUA of 0.7 mg/dl and carried a homozygous *SLC22A12*: p.E458K. For these six mutations, both the allelic and genotypic distributions differed significantly between the hypouricemia and normal controls (*p* < 0.003). All these mutations did not appear in normal controls or HUA cohort and had no significant effects on HUA susceptibility (*p* > 0.003). The remaining rare mutations were sparsely detected in normal control or HUA subject and presented no significance (*p* > 0.003). Further, we performed gene‐based association analyses in which individuals carrying any rare mutation were set as gene mutation carriers. The ratio of rare mutation carriers in the hypouricemia group was significantly higher than that in normal controls (19.3% vs. 2.14%, OR = 10.96, *p* = 3.19E^‐7^), while aggregated rare mutations conferred no significant effects on HUA susceptibility (0.69% vs. 2.14%, OR = 0.32, *p* = 0.14). Unexpectedly, two previously reported hypouricemia causative mutations *SLC22A12*: p.R90H and *SLC22A12*: p.W258X were found in three normal controls rather than in the hypouricemia individual (Table 3).

**Table 3:** The hypouricemic individuals with rare mutations and the normal controls with reported hypouricemia causative mutations

Individuals	Gender	SUA (mg/dl)	Variants	Variants status	Novel or not
QQY23	Male	0.63	*SLC2A9*: c.G62A/p.T21I	Heterozygous	rs748372830
17QD5230	Female	1.57	*SLC2A9*: c.C38T/p.G13D	Heterozygous	Novel
QLY630	Male	1.92	*SLC22A12*: c.149delG/p.W50fs	Heterozygous	rs752156476
N2401	Female	1.93	*SLC22A12*: c.149delG/p.W50fs	Heterozygous	rs752156476
17QD3912	Female	1.77	*SLC22A12*: c.A1145T/p.Q382L	Compound heterozygous	rs765990518
			*SLC22A12*: c.G1639C/p.V547L		Novel
17QD2146	Male	0.70	*SLC22A12*: c.G1372A/p.E458K	Homozygous	rs747742344
QLY2219	Male	5.63	*SLC22A12*: c.G269A/p.R90H	Heterozygous	rs121907896
ZY26‐183	Female	4.55	*SLC22A12*: c.G269A/p.R90H	Heterozygous	rs121907896
QLY2255	Male	5.17	*SLC22A12*: c.G774A/p.W258X	Heterozygous	rs121907892

Note

Variants were expressed in two forms of nucleotide change and amino acid change according to reference sequences of *SLC2A9* (NM_020041.3; NP_064425.2) and *SLC22A12* (NM_144585.4; NP_653186.2).

To convert SUA in mg/dl to μmol/l, multiply by 60.

Abbreviation: SUA, serum uric acid.

Separate analyses for male and female are displayed in Table S1 and Table S2. p.T21I in*SLC2A9*, p.W50fs and p.E458K in *SLC22A12* were significantly associated with hypouricemia in male samples whereas p.G13D in*SLC2A9*, p.W50fs, p.Q382L and p.V547L in *SLC22A12* were significantly associated with hypouricemia in females.

### 3.4 Pathogenicity predictions for the nonsynonymous mutations

Annotations by Annovar were conducted to evaluate the variant pathogenicity. As shown in Data S2, the six mutations susceptible to hypouricmia were not completely consistent with the predicted pathogenicity by software. For instance, *SLC22A12*: p.E458K and *SLC22A12*: p.Q382L conformed to “deleterious” as predicted by the software while *SLC2A9*: p.T21I, *SLC2A9*: p.G13D and *SLC22A12*: p.V547L were predicted to be benign. Additionally, we predicted the conserved domains of the two urate transporters by pasting the whole protein sequences to NCBI (https://www.ncbi.nlm.nih.gov/Structure/cdd/wrpsb.cgi). Q382 in *SLC22A12* was conserved while other loci were not. *SLC22A12*: p.W50fs was a truncated protein with premature codon termination at amino acid 64 and deemed as a loss‐of‐function mutation (Li et al., 2013).

## 4 DISCUSSION

The main findings in this study was that six rare SNVs p.T21I and p.G13D in *SLC2A9*, p.W50fs, p.Q382L, p.V547L and p.E458K in *SLC22A12*, occurred in six hypouricemia subjects. Both single locus and gene‐based association analyses further corroborated the hypouricemia susceptibility from a statistical perspective. Among them, *SLC2A9*: p.G13D and *SLC22A12*: p.V547L were newly reported.

To date, most reported hRHUC patients were of Japanese origin and harbored stopgain mutation *SLC22A12*: p.W258X, among which the homozygous carriers had much higher risk for developing acute kidney events (Ichida et al., 2004; Zhou et al., 2018). hRHUC patients were reported less in Korea but a majority carried *SLC22A12*: p.W258X as well (Zhou et al., 2018). Intriguingly, people with heterozygous W258X mutation had SUA measurement ranging from content to hypouricemia criterion (Ichida et al., 2004) to normal range (Iwai et al., 2004; Taniguchi et al., 2005) and ~3% of alleles occurred with this mutation in 1875 subjects from an epidemiological survey which represented the general population in Japan (Iwai et al., 2004), suggesting the harmlessness of the heterozygote to the general population. Not surprisingly, the *SLC22A12*: p.W258X mutation showed a protective effect against gout incidence in comparison with healthy controls (Taniguchi et al., 2005). As in Caucasians, Israel–Arab, Iraqi jews (Zhou et al., 2018), Pakistan (Jeannin et al., 2014) and India (Chakraborty & Sural, 2013), scattered hypouricemia cases have also been reported in China which displayed dispersed mutation spectrum. For example, homozygous *SLC22A12*: p.R90H was found in two brothers with hypouricemia (Yan, Cheng, Chen, & Lin, 2010), compound heterozygous *SLC22A12:* p.P78L plus p.Q382L (Lam et al., 2008), homozygous *SLC2A9:* p.W238X (Shen et al., 2014), homozygous splicing mutation c.1215+1 G>A in *SLC2A9* (Mou, Jiang, & Hu, 2015), and compound heterozygous *SLC22A12:* p.R90H plus p.M430fsX466 (Zhou et al., 2018) were found in each hypouricemia patient, respectively. In another literature, three hypouricemia siblings and their normal father had heterozygous *SLC22A12:* p.A51fsX64 (Li et al., 2013). In fact, two siblings were within low normal range with SUA being 2.6 mg/dl and 2.0 mg/dl, respectively. In the second family, the hypouricemia patient had compound heterozygous *SLC22A12:* p.T217M plus *SLC2A9:* p.P516T but his affected mother only had one heterozygous *SLC22A12:* p.T217M (Li et al., 2013). To summarize, *SLC2A9:* p.R90H allele accounted for 27.8% (5/9*2) among the hypouricemia patients which was much greater than that in Japan and Korea (Zhou et al., 2018), while W258X was not detected in any hypouricemia patient in Chinese samples. The *SLC22A12* mutants were of major subtype responsibility for hRHUC, which was identical in varied ethnicities (Claverie‐Martin et al., 2018; Zhou et al., 2018). The high incidence of hRHUC1 (OMIM #220150) has been reported in the Asian region and is attributed to the high frequency of the p.W258X (2.30%–2.37%) and p.R90H (0.40%) in *SLC22A12* among Japanese (Iwai et al., 2004; Taniguchi et al., 2005) and general Korean populations (Lee et al., 2008), which is indicative of a founder mutation in the Asian continent. As for the Roman (the largest and the most widespread ethnic minority of Europe), the high frequency of *SLC22A12* variants causing hRHUC1 may be due to the high frequency of the p.L415_G417del (1.87%–1.92%) and p.T467M (5.56%) dysfunctional variants in the Roma general population (Gabrikova, Bernasovska, Sokolova, & Stiburkova, 2015; Stiburkova et al., 2016). In this study, we still did not detect W258X mutation in hypouricemia patients, but one heterozygote occurrence was observed in one normal person unexpectedly. The prevalence of W258X mutation was 0.18% (1/280*2) in our normal cohort which was much lower than that in the Japanese cohort (Iwai et al., 2004; Taniguchi et al., 2005), suggesting the genetic heterogeneity between the two populations. In this study, the *SLC22A12*: p.R90H was also not detected in any hypouricemia subject, but was detected in heterozygote occurrences in two normal controls.

Notably, only six hypouricemia subjects were identified with rare nonsynonymous mutations, of which the detection rate (6/31) was much lower than other studies. For example, 30/32 patients were detected with homozygous, compound heterozygous or heterozygous mutations in *SLC22A12* in one single cohort (Ichida et al., 2004). Our hypouricemia cohort was selected from a community‐based database where the biochemical values were examined once in field survey. A few hypouricemia samples may be temporary or secondary to other diseases rather than persistent or primary hypouricemia. Some hypouricemia samples may be hereditary xanthinuria resulting from inherited deficiency of xanthine oxidorectase and aldehyde oxidase (Mraz et al., 2015), proportion would not be substantial in terms of rare report in Chinese (Zhou et al., 2015). Thus, we have reason to justify that most hypouricemia samples can be diagnosed as hRHUC. The primary explanation for such a low detection rate should be that the genetic variation for hRHUC may not only locate in these two genes in the Chinese population since our sequence depths on the exon regions of the two genes were yielded 1,035‐fold on average which was enough to discriminate germline mutations. Therefore, further whole‐genome studies to reveal more causal genes and mutations are suggested. In sex‐specific analysis, some of variants failed to reach statistical significance. Sample size might be one of the major reasons for the negative results. Besides, noting that some previously identified hypouricemia causal variants were found in normouricemia (Iwai et al., 2004; Taniguchi et al., 2005) as with ours, that the six SNVs are not necessarily causal of hypouricemia could not be excluded and it is possible that at least some of the six SNVs might be false positives. A larger sample size and further evidence from functional experiments would be necessary to replicate the associations and to confirm their urate transportation activity and pathophysiological role on hypouricemia.

For the common variant rs3733591, the C allele had been found to increase risk of higher SUA, gout and tophi (Hollis‐Moffatt et al., 2011; Tu et al., 2010). However, in this study, rs3733591 did not reach HWE and was excluded from association analysis. For the common variant rs16890979, C allele was associated with higher SUA and gout risk (Dehghan et al., 2008). The present study confirmed the result from another aspect: T allele conferred substantial risk for hypouricemia (OR >1) although the association only showed a marginal strength. Considering the relatively small sample size in this study, the negative result can be explained by the insufficient statistical power. For the other widely reported common variants rs2280205, rs6820230 and rs2276961, no significant effect on hypouricemia or HUA was identified in our samples, which was in accordance with previous reports (Hurba et al., 2014; Xing et al., 2015). As for the rare variants, both single locus and aggregation analysis confirmed increased risk for hypouricemia although they had no significant protection against HUA. These results imply that rare variants have greater impact on urate transportation and may serve as potential targets for urate transporter blocker, which is substantiated by the recent whole‐exome sequencing association studies (Tin et al., 2018).

Additionally, we arranged hypouricemia samples according to SUA value and two SUA sections were concentrated with the first at 0.3–1.0 mg/dl and the second at 1.7–2.0 mg/dl. We suspect that those with lower SUA value more likely carry homozygous mutations or compound heterozygous mutations and those with relatively higher SUA level may result from heterozygous mutations. However, the degree of hypouricemia was not entirely consistent with mutation type as suspected. For example, patient 1 encoded with QQY23 had SUA of 0.63 mg/dl but carried a heterozygous *SLC2A9:* p.Thr21Ile. Patient 5 encoded with 17QD3912 had SUA of 1.77 mg/dl but carried compound heterozygous *SLC22A12*: p.Gln382Leu and *SLC22A12*: p.Val547Leu. Patient 6 encoded with 17QD2146 had SUA of 0.7 mg/dl and carried a homozygous *SLC22A12*: p.Glu458Lys, which was in line with our assumptions. *SLC2A9*: p.Gly13Asp and *SLC22A12*: p.Val547Leu were first reported but tended to be nonpathogenic by software prediction. As for* SLC22A12*: p.Gln382Leu and *SLC22A12*: p.Glu458Lys, both mutations were predicted to be pathogenic. *SLC22A12*: p.Gln382Leu was recognized as conservative as well as likely pathogenic in ClinVar, which was in accordance with functional characterization (Wakida et al., 2005). Besides, the phenotypic severity of hRHUC is not necessarily correlated with function status of the mutants (Mancikova et al., 2016) and some patients even occur nonrenal complications such as rhabdomyolysis (Chakraborty & Sural, 2013) and neurological symptoms of posterior reversible encephalopathy syndrome (Fujinaga et al., 2013; Mou et al., 2015; Shima et al., 2011), which altogether implies a complexity of genetic involvement and pathophysiological process underlying the disease spectrum. Primary hypouricemia is a common characteristic of xanthine dehydrogenase (XDH) deficiency (Xanthinuria 1, OMIM #278300) and reduced synthesis of molybdenum cofactor (Xanthinuria 2, OMIM #603592) and hRHUC1 (OMIM #220150) and hRHUC2 (OMIM #612076). However, in some of these patients neurological symptoms were not observed, including cases with extremely low SUA values near 0 (Stiburkova, Ichida, & Sebesta, 2011; Stiburkova et al., 2012; Stiburkova, Pavelcova, Petru, & Krijt, 2018). These discrepant reports suggest that the protective systems involving plasma uric acid may not be essential. The relationship between SUA and neuroprotective action remains a debated issue which needs more well‐designed prospective studies and functional research in the future. Unluckily, information on past medical history could not be obtained from this database to estimate the relationship between hypourcemia and other phenotypes.

In conclusion, our amplicon targeted sequencing and statistical analyses identified six rare nonsynonymous variants associated with hypouricemia in the Han Chinese population. Functional study together with comprehensive phenotype‐genotype research should be necessary to reveal the exact involvement of the variants in urate transportation and disease spectrum. Whole‐genome screen to reveal the new causal genes and mutations for hRHUC in Han Chinese samples are suggested.

## CONFLICT OF INTEREST

None declared.

## Data Availability

The datasets used and analyzed in the current study are available from the corresponding author on reasonable request.
