# Supplementary material for: Amplicon targeted resequencing for SLC2A9 and SLC22A12 identified novel mutations in hypouricemia subjects
Source: Mol Genet Genomic Med. 2019 May 26;7(7):e00722. doi: 10.1002/mgg3.722 (PMC6625124; doi:10.1002/mgg3.722)
Supplement: Supplementary file 1 [file MGG3-7-e00722-s001.docx]

Supplementary Table 1 Association results of hypouricemia (1) versus normal control (2) and hyperuricemia (3) versus normal control (2) in male

| CHR | BP | Variants | Allele | (1) | (2) | (3) | (1) Vs (2) OR | (1) Vs (2) p | (3) Vs (2) OR | (3) Vs (2) p | Novel or not |
| --- | --- | --- | --- | --- | --- | --- | --- | --- | --- | --- | --- |
| chr4 | 9909923 | c.G1049A/p.P350L | G | 25(0.735) | 209(0.746) | 207(0.719) | 1.060 | 0.8881 | 1.152 | 0.4563 | rs2280205 |
|  |  |  | A | 9(0.265) | 71(0.254) | 81(0.281) | 0.47~2.38 |  | 0.79~1.67 |  |  |
| chr4 | 9922167 | c.C844T/p.V282I | C | 33(0.971) | 275(0.982) | 285(0.990) | 1.667 | 0.6422 | 0.579 | 0.4519 | rs16890979 |
|  |  |  | T | 1(0.029) | 5(0.018) | 3(0.010) | 0.19~14.29 |  | 0.14~2.44 |  |  |
| chr4 | 10020615 | c.A233G/p.V78A | A | 34(1.000) | 279(0.996) | 287(0.997) | - | 0.7271 | 0.972 | 0.9841 | rs907584363 |
|  |  |  | G | 0(0.000) | 1(0.004) | 1(0.003) |  |  | 0.06~16.67 |  |  |
| chr4 | 10020618 | c.A230C/p.V77G | A | 34(1.000) | 280(1.000) | 287(0.997) | - | - | - | 0.3237 | rs183263293 |
|  |  |  | C | 0(0.000) | 0(0.000) | 1(0.003) |  |  |  |  |  |
| chr4 | 10022981 | c.C73T/p.G25R | C | 17(0.500) | 145(0.518) | 153(0.531) | 1.074 | 0.8440 | 0.948 | 0.7493 | rs2276961 |
|  |  |  | T | 17(0.500) | 135(0.482) | 135(0.469) | 0.53~2.17 |  | 0.68~1.32 |  |  |
| chr4 | 10022992 | c.G62A/p.T21I | A | 1(0.029) | 0(0.000) | 0(0.000) | - | **0.0041** | - | - | rs748372830 |
|  |  |  | G | 33(0.971) | 280(1.000) | 288(1.000) |  |  |  |  |  |
| chr4 | 10027542 | c.C49T/p.A17T * | C | 32(0.941) | 262(0.936) | 264(0.917) | 0.91 | 0.9020 | 1.323 | 0.3858 | rs6820230 |
|  |  |  | T | 2(0.059) | 18(0.064) | 24(0.083) | 0.20~4.17 |  | 0.70~2.5 |  |  |
| chr11 | 64359297 | c.G269A/p.R90H | G | 34(1.000) | 279(0.996) | 288(1.000) | - | 0.7271 | - | 0.3101 | rs121907896 |
|  |  |  | A | 0(0.000) | 1(0.004) | 0(0.000) |  |  |  |  |  |
| chr11 | 64360303 | c.A455G/p.Y152C | A | 34(1.000) | 279(0.996) | 288(1.000) | - | 0.7271 | - | 0.3101 | Novel |
|  |  |  | G | 0(0.000) | 1(0.004) | 0(0.000) |  |  |  |  |  |
| chr11 | 64361219 | c.G774A/p.W258X | G | 34(1.000) | 279(0.996) | 288(1.000) | - | 0.7271 | - | 0.3101 | rs121907892 |
|  |  |  | A | 0(0.000) | 1(0.004) | 0(0.000) |  |  |  |  |  |
| chr11 | 64367925 | c.G1372A/p.E458K | G | 32(0.941) | 280(1.000) | 288(1.000) | - | **4.72E^-05^** | - | - | rs747742344 |
|  |  |  | A | 2(0.059) | 0(0.000) | 0(0.000) |  |  |  |  |  |
| chr11 | 64359177 | c.149delG/p.W50fs | G | 33(0.971) | 280(1.000) | 288(1.000) | - | **0.0041** | - | - | rs752156476 |
|  |  |  | delG | 1(0.029) | 0(0.000) | 0(0.000) |  |  |  |  |  |

CHR, chromosome; BP, base position; OR, odds ratio; “-” means uncalculated; P < 0.0042 (0.05/12) as statistical significance and significant p-values in bold. BP was determined by reference sequences of S*LC2A9* (NC_000004.11, region: 9827848…10041872) and *SLC22A12* (NC_000011.9, region: 64358113…64369825) and variants were expressed in two forms of nucleotide change and amino acid change according to reference sequences of *SLC2A9* (NM_020041.3; NP_064425.2) and *SLC22A12* (NM_144585.4; NP_653186.2). * denotes the variant annotated by *SLC2A9* (NM_001001290.1; NP_001001290.1). For each variant, the allele in the first line was the reference allele and the second line was the altered allele that the reported OR correlates with.

Supplementary Table 2 Association results of hypouricemia (1) versus normal control (2) and hyperuricemia (3) versus normal control (2) in female

| CHR | BP | Variants | Allele | (1) | (2) | (3) | (1) Vs (2) OR | (1) Vs (2) p | (3) Vs (2) OR | (3) Vs (2) p | Novel or not |
| --- | --- | --- | --- | --- | --- | --- | --- | --- | --- | --- | --- |
| chr4 | 9909923 | c.G1049A/p.P350L | G | 20(0.714) | 193(0.689) | 202(0.701) | 0.887 | 0.7848 | 0.944 | 0.7540 | rs2280205 |
|  |  |  | A | 8(0.286) | 87(0.311) | 86(0.299) | 0.38~2.09 |  | 0.66~1.35 |  |  |
| chr4 | 9922167 | c.C844T/ p.V282I | C | 26(0.929) | 277(0.989) | 285(0.990) | 7.092 | 0.0154 | 0.972 | 0.9723 | rs16890979 |
|  |  |  | T | 2(0.071) | 3(0.011) | 3(0.010) | 1.14~50 |  | 0.19~4.76 |  |  |
| chr4 | 10022981 | c.C73T/p.G25R | C | 18(0.643) | 132(0.471) | 135(0.469) | 0.496 | 0.0836 | 1.011 | 0.9490 | rs2276961 |
|  |  |  | T | 10(0.357) | 148(0.529) | 153(0.531) | 0.22~1.11 |  | 0.72~1.41 |  |  |
| chr4 | 10023016 | c.C38T/p.G13D | C | 27(0.964) | 280(1.000) | 288(1.000) | - | **0.0015** | - | - | Novel |
|  |  |  | T | 1(0.036) | 0(0.000) | 0(0.000) |  |  |  |  |  |
| chr4 | 10027542 | c.C49T/p.A17T * | C | 26(0.929) | 257(0.918) | 269(0.934) | 0.860 | 0.8431 | 0.789 | 0.4616 | rs6820230 |
|  |  |  | T | 2(0.071) | 23(0.082) | 19(0.066) | 0.19~3.85 |  | 0.42~1.49 |  |  |
| chr11 | 64359297 | c.G269A/p.R90H | G | 28(1.000) | 279(0.996) | 288(1.000) | - | 0.7514 | - | 0.3101 | rs121907896 |
|  |  |  | A | 0(0.000) | 1(0.004) | 0(0.000) |  |  |  |  |  |
| chr11 | 64360355 | c.506+1G>A | G | 28(1.000) | 280(1.000) | 287(0.997) | - | - | - | 0.3237 | rs58174038 |
|  |  |  | A | 0(0.000) | 0(0.000) | 1(0.003) |  |  |  |  |  |
| chr11 | 64361134 | c.G689A/p.R230Q | G | 28(1.000) | 279(0.996) | 288(1.000) | - | 0.7514 | - | 0.3101 | rs759297223 |
|  |  |  | A | 0(0.000) | 1(0.004) | 0(0.000) |  |  |  |  |  |
| chr11 | 64359177 | c.149delG/p.W50fs | G | 27(0.964) | 280(1.000) | 288(1.000) | - | **0.0015** | - | - | rs752156476 |
|  |  |  | delG | 1(0.036) | 0(0.000) | 0(0.000) |  |  |  |  |  |
| chr11 | 64367222 | c.A1145T/p.Q382L | A | 27(0.964) | 280(1.000) | 288(1.000) | - | **0.0015** | - | - | rs765990518 |
|  |  |  | T | 1(0.036) | 0(0.000) | 0(0.000) |  |  |  |  |  |
| chr11 | 64369000 | c.G1639C/p.V547L | C | 1(0.036) | 0(0.000) | 0(0.000) | - | **0.0015** | - | - | Novel |
|  |  |  | G | 27(0.964) | 280(1.000) | 288(1.000) |  |  |  |  |  |

CHR, chromosome; BP, base position; OR, odds ratio; “-” means uncalculated; P < 0.0045 (0.05/11) as statistical significance and significant p-values in bold. BP was determined by reference sequences of S*LC2A9* (NC_000004.11, region: 9827848…10041872) and *SLC22A12* (NC_000011.9, region: 64358113…64369825) and variants were expressed in two forms of nucleotide change and amino acid change according to reference sequences of *SLC2A9* (NM_020041.3; NP_064425.2) and *SLC22A12* (NM_144585.4; NP_653186.2). * denotes the variant annotated by *SLC2A9* (NM_001001290.1; NP_001001290.1). For each variant, the allele in the first line was the reference allele and the second line was the altered allele that the reported OR correlates with.
